# Chemerin and Polycystic Ovary Syndrome: A Comprehensive Review of Its Role as a Biomarker and Therapeutic Target

**DOI:** 10.3390/biomedicines12122859

**Published:** 2024-12-16

**Authors:** Stefano Palomba, Giuseppe Seminara, Flavia Costanzi, Donatella Caserta, Antonio Aversa

**Affiliations:** 1 Unit of Obstetrics and Gynecology, Grande Ospedale Metropolitano of Reggio Calabria, University Sapienza of Rome, 89124 Reggio, Italy; 2 Department of Experimental and Clinical Medicine, University “Magna Graecia” of Catanzaro, 88100 Catanzaro, Italy; dott.giuseppeseminara@gmail.com (G.S.); aversa@unicz.it (A.A.); 3 Unit of Gynecology, Sant’Andrea Hospital of Rome, University Sapienza of Rome, 00185 Roma, Italy; costanzi.flavia@gmail.com (F.C.); donatella.caserta@uniroma1.it (D.C.)

**Keywords:** chemerin, hyperandrogenism, infertility, insulin resistance, obesity, polycystic ovary syndrome (PCOS), pregnancy, ovarian dysfunction

## Abstract

**Background:** Chemerin, an adipokine implicated in inflammatory, metabolic, and adipogenic processes, has been detected in high serum concentration in women with polycystic ovary syndrome (PCOS) and seems to play a role in PCOS pathogenesis. Moreover, at present, no comprehensive and critical document is available in the literature on this topic. The aim of the current study was to comprehensively review the latest available data to confirm the evidence about the association between chemerin and PCOS, highlighting its potential role as an upcoming biomarker and therapeutic target. **Methods:** A search in the literature of studies published between 2019 and 2024 was conducted using PubMed, Cochrane Library, and Web of Science, focusing on research related to chemerin, PCOS, and PCOS-related features, comorbidities, and complications. A qualitative structured synthesis of key findings was performed according to the specific thematic areas selected, including and discussing clinical data on women with PCOS and experimental studies in humans and animal models of PCOS. **Results:** Available data confirm increased serum levels of chemerin in women with PCOS compared with controls, independent of obesity and body mass index. Chemerin is associated with insulin resistance, hyperandrogenism, and ovarian dysfunction in PCOS individuals, inhibiting folliculogenesis and steroidogenesis. Experimental animal models underscore chemerin’s regulatory roles through its receptors within the hypothalamic–pituitary–ovarian axis and peripheral tissues. High systemic levels of chemerin in PCOS may also be related to the increased risk of pregnancy complications, especially gestational diabetes mellitus and preeclampsia. **Conclusions:** The current review study highlights the role of chemerin in PCOS pathophysiology, severity, and associated comorbidities and complications, assessing its value as a future biomarker and foreshadowing its potential as a therapeutic target.

## 1. Introduction

Polycystic ovary syndrome (PCOS) is the most prevalent reproductive disorder in women, with a multifactorial etiology with genetic, metabolic, and environmental factors [1]. PCOS is a cluster of clinical and metabolic abnormalities, manifested as hyperandrogenism, hirsutism, anovulation, polycystic ovarian morphology (PCOM), inflammation, insulin resistance (IR), glucose intolerance, obesity, and metabolic syndrome [2,3,4]. Additionally, PCOS is strongly associated with increased risk of cardiovascular disease [5,6], eating disorders [7], psychological/psychiatric alterations [8], infertility [9] and pregnancy complications [10]. A significant socioeconomic burden is secondary to PCOS diagnosis [11,12,13].

PCOS diagnostic criteria were defined during the Rotterdam consensus workshop in 2003 [14] and confirmed by subsequent expert opinions and clinical guidelines [15,16,17]. Recently, international guidelines [18] underlined to use these same criteria with few clinical and practical changes, including the definition of ovarian dysfunction and the anti-Mullerian hormone (AMH) assay. In brief, PCOS diagnosis requires the finding of at least two of the following three features: ovulatory dysfunction (defined as irregular menses), hyperandrogenism, and PCOM at ultrasound [18]. These standardized criteria are intended to encompass the heterogeneous presentations of PCOS, although controversy continues over phenotypic classification [19].

One of the more studied alterations present in PCOS patients is adipose tissue dysfunction. A substantial number of studies have shown that obesity is more prevalent in women with PCOS than in those without the syndrome, and obesity appears to worsen metabolic derangements [20]. The dysfunction of adipose tissue includes alterations of the adipokine profiles that may play a role in the physiopathology of the syndrome [21]. For example, leptin and adiponectin are significantly modulated in PCOS, with a potential role in the metabolic and inflammatory components of the syndrome [22]. In addition, the abnormalities in adipokine levels may be considered suitable biomarkers to assess the metabolic risk in PCOS patients, suggesting the presence of a strong association between metabolic and endocrine alterations [23].

Chemerin is a novel and ubiquitous adipokine [24]. It has been detected in different species (humans, rodents, bovines), and it is expressed mostly in white adipose tissues, the liver, and the placenta, but also in brown adipose tissue, the lungs, the kidneys, the ovaries, skeletal muscle, and the heart [25]. It is involved in inflammation, energy metabolism, adipogenesis, angiogenesis, and insulin secretion in the adipose cells and ovaries [26]. Chemerin receptors are protein G-coupled receptors. Chemokine-like receptor 1 (CMKLR1) is expressed in monocyte-derived dendritic cells and macrophages, even if its messenger ribonucleic acid (mRNA) has been detected in various tissues such as hematopoietic tissues, adipocytes, endothelial cells, osteoclasts, and ovarian cells [27,28]. It has a role in adipogenesis and adipocyte maturation, promotes phagocytosis, and enhances insulin signaling [29,30,31]. A second receptor, C-C motif chemokine receptor-like 2 (CCRL2), has been identified in human neutrophils, macrophages, mast cells, lung epithelial cells, and ovaries [28]. This receptor might present chemerin to nearby CMKLR1-positive cells to regulate immune responses and inflammation [32]. Finally, another receptor, the G protein receptor 1 (GPR1), has also been identified in humans and rodents, and it is involved in brain peptide transmission [33]. Contrarily to CMKLR1 and CCRL2, this receptor has not been found in immune cells but in central nervous system cells, murine brown adipose tissue, white adipose tissue, and skeletal muscle [28]. It is an active chemerin receptor and regulates glucose homeostasis and, in the mouse ovary, progesterone secretion during the follicular development, corpus luteum formation, and luteolysis [34].

The existence of a correlation between chemerin and PCOS has been repeatedly evoked, and several studies have outlined chemerin among the adipokines with a potential pathogenic role in PCOS [23]. Specifically, chemerin levels appear to be related to various mechanisms involved in PCOS pathogenesis, such as obesity, hyperinsulinism, low-grade inflammation, or reduced ovarian steroidogenesis [23]. Elevated serum and ovarian levels of chemerin have been initially shown in dihydrotestosterone (DHT)-induced rat PCOS models [35], and recent systematic reviews with meta-analyses have confirmed the close association between serum chemerin levels and PCOS diagnosis [20,36,37,38].

Based on these considerations, herein we present a comprehensive review of the latest data aimed to address the roles played by the chemerin system in women with PCOS, with particular regard for PCOS-related features, comorbidities, and complications, to consolidate evidence about the association between chemerin and PCOS and to highlight its potential role as an upcoming biomarker and therapeutic target of the syndrome.

## 2. Methods

An extensive literature search was performed for articles from 2019 to October 2024 using PubMed, the Cochrane Library, and the Web of Science database. Specific keywords used in the search were: “chemerin” and “polycystic ovary syndrome” or “polycystic ovary disease” or “PCOS”. Subsequently, the same search term (“chemerin”) was paired with terms covering specific features/characteristics related to PCOS including “polycystic ovaries”, “polycystic ovarian morphology”, “oligo-amenorrhea”, “oligo-anovulation”, “ovulatory dysfunction”, “chronic anovulation”, “amenorrhea”, “irregular menses”, “hyperandrogenism”, “testosterone”, “anti-Mullerian hormone”, “AMH”, “hyperestrogenism”, “estrogens”, “estradiol”, “hyperinsulinemia”, “insulin resistance”, “hirsutism”, “inflammation”, “body mass index”, “BMI”, “obesity”, “infertility”, “sterility”, “diabetes”, “autoimmunity”, “pregnancy complication”, and “placenta”. A specific search was also performed using “chemerin” and “gestational diabetes mellitus”, “pregnancy complications”, and “preeclampsia”.

Two independent reviewers (G.S., F.C.) screened the titles and abstracts of all selected articles, focusing on research related to chemerin, PCOS, and related features, comorbidities, and complications. No specific inclusion or exclusion criteria were initially applied at the study design. The full texts of the selected articles were revised, and incongruities were resolved by a third reviewer (S.P.) to confirm consistency in study selection. Citation tracking was also performed on the bibliographies of articles selected for review, which identified additional relevant literature. Studies were included in this review only if they were considered scientifically relevant. Clinical data specific for women with PCOS were considered first. When specific data were lacking, results from general population studies were reported, especially in the presence of PCOS-related characteristics (such as hyperandrogenism, obesity, IR) or similar comorbidities/complications. Experimental studies on humans or on animal models of PCOS or with PCOS features were also included and discussed.

The main thematic areas of interest were identified, and data were tabulated, summarized, and discussed for each specific area. Data were analyzed qualitatively and presented in a narrative and structured fashion. No attempt to perform a quantitative analysis was planned.

## 3. Results

The following thematic areas of interest were identified and discussed: obesity, IR, hyperandrogenism, ovarian dysfunction, infertility, and pregnancy complications, the latter including gestational diabetes mellitus (GDM) and preeclampsia (PE). The main clinical studies on these correlations are summarized in Table 1, Table 2, Table 3, Table 4 and Table 5. Available interventional data were also intercepted and are detailed in different subsections. Table 6 details the characteristics of these interventional studies.

### 3.1. Chemerin and Obesity in PCOS

Obesity is a common comorbidity in PCOS and significantly worsens the metabolic and reproductive consequences of this disease [1]. When compared with non-PCOS controls, obese patients with PCOS are at higher risk for IR, hyperinsulinemia, and type 2 diabetes (T2D), which exacerbate androgen excess and menstrual irregularities [20,84] and CVD risk [85]. Several experimental data have been produced to study the association between chemerin and obesity in women with PCOS and to explain the potential influence of chemerin in PCOS on body weight and on the adipose tissue, and vice versa.

To investigate adipokine ovarian expression in PCOS women in comparison with controls and women with PCOM, a case–control study was conducted on women undergoing an in vitro fertilization (IVF) procedure, dosing follicular fluid (FF) concentration and human granulosa cell (hGC) mRNA expression of adipokines and their receptors [40]. Chemerin expression both in FF and hGCs was greater in obese women than in normal-weight women [40]. FF chemerin concentration was higher in normal-weight PCOS women than in body mass index (BMI)-matched controls and PCOM women. hGC mRNA levels were higher in the obese PCOS group than in the PCOM one [40]. Chemerin follicular concentration positively correlated with chemerin mRNA levels in hGCs, and both strongly positively correlated with BMI [40]. Contrarily to chemerin, mRNA levels of CMKLR1 were almost undetectable in obese women. CMKLR1 expression was predominant in women with PCOS, and CMKLR1 mRNA levels were negatively correlated with BMI and chemerin levels [40]. CCRL2 expression varied only according to BMI, whereas GPR1 expression did not change in any condition [40]. These data seem to suggest that FF chemerin and CMKLR1 expression were increased in PCOS regardless of BMI. However, the co-occurrence of obesity may suppress the expression of CMKLR1 in hGCs, thus leading to a higher resistance to chemerin action [40].

A study involving a series of nonobese and obese women with and without PCOS evaluated the circulating levels of adipokines during fasting and after glucose, lipid, and protein challenges [39]. A significant association between obesity and serum chemerin levels at fasting was found. However, no significant association was reported between PCOS and chemerin [39]. Another study confirmed an increased concentration of chemerin levels in PCOS patients as compared with controls independently of the presence of obesity or metabolic syndrome [47]. In 2021, two different meta-analyses reached similar conclusions, documenting a significant association between PCOS and chemerin independent of BMI, but with chemerin serum levels tending to be higher in overweight and obese patients [36,37].

However, the effect of BMI or obesity on serum chemerin levels in women with PCOS is still under discussion. In fact, more recent studies did not provide unequivocal results either. A case–control study involving PCOS women and body weight- and BMI-matched controls confirmed that levels of chemerin were significantly higher in PCOS than in non-PCOS controls, regardless of BMI. Indeed, among PCOS patients, serum chemerin level was higher in normal-weight and overweight cases compared with the normal-weight group, even if the difference did not achieve the statistical significance [42]. On the other hand, in another study, mean serum chemerin levels were found significantly higher in the obese PCOS group compared with the lean PCOS group, but there was no significant difference in serum chemerin levels between lean women with and without PCOS [45].

Recently, a study investigated the effects of abnormal body weight (and tobacco smoke exposure) on circulating chemerin levels in women with PCOS [41]. Although no significant differences in chemerin concentrations compared with controls were found, in the PCOS group, chemerin was significantly higher among overweight and obese subjects in comparison with normal-weight PCOS patients [41]. Interestingly, visceral obesity was not associated with significant changes in chemerin concentrations. Chemerin concentration was positively correlated with BMI and waist to hip ratio (WHR), as well as with the concentrations of triglycerides and fasting glucose, but negatively correlated with high-density lipoprotein cholesterol (HDL-C) levels [41]. In the group of smoking women with PCOS, the most significant correlation among all examined adipokines was with chemerin [41]. Thus, chemerin in women with PCOS could affect the metabolic status and hyperandrogenism, and exposure to tobacco smoke could be an additional factor influencing these relationships [41].

To find an option to decrease serum chemerin levels in obese PCOS patients, a randomized controlled trial (RCT) studied the effect of caloric restriction and 5 g/day supplementation with thylakoid-rich spinach extract, a potential dietary supplement for the reduction of appetite [83]. The thylakoid group had reduced fat mass and chemerin levels compared with the placebo group, thus leading to a hypothesis to recommend this supplementation for obese women with PCOS [83].

In conclusion, in the PCOS population, patients with higher BMI tend to have higher chemerin levels than normal-weight patients. However, obesity seems to do not influence the serum chemerin levels in PCOS.

### 3.2. Chemerin and IR in PCOS

Up to 70% of all women with PCOS have IR, and this feature exists irrespective of obesity [86]. IR leads to compensatory hyperinsulinemia that further amplifies androgen overproduction and impairs ovarian function, aggravating the reproductive and metabolic manifestations of PCOS [2,87].

Based on the hypothesis that chemerin may contribute to perturbed glucose metabolism in PCOS and worsen PCOS severity, a single study [44] investigated the relationship between IR of the hGCs with chemerin levels in the FF and chemerin effects on glucose metabolism in hGCs in PCOS patients [44]. The concentrations of chemerin in the FF and plasma were significantly elevated in PCOS patients with IR in comparison with the PCOS without IR, non-PCOS with IR, and non-PCOS without IR groups [44]. The concentration of chemerin in the FF was positively correlated with the concentration of insulin in the FF [44]. Moreover, pretreatment of hGCs with chemerin (100 ng/mL) for 24 h significantly unbalanced insulin signaling, enhancing insulin-stimulated insulin receptor substrate (IRS) 1 Ser307 phosphorylation, and attenuated insulin-stimulated phosphorylation of IRS 1/2 Tyr612 and Akt Ser473 [44]. Chemerin pretreatment significantly diminished total glucose transporter (GLUT)-4 expression and attenuated insulin-induced GLUT4 translocation to the membrane, weakening the glucose uptake ability [44]. The short interfering ribonucleic acid (siRNA)-mediated knockdown of CMKLR1 reversed these chemerin-induced effects [44]. These findings suggest an effect of chemerin on impairment of the insulin signaling pathway. Chemerin-induced IR may be further amplified by elevated insulin in FF from PCOS patients with IR because of the induction of chemerin expression by insulin [44].

Data on the relationship between serum chemerin levels and surrogate markers of IR are partially controversial and conflicting. In fact, increased serum chemerin values in PCOS patients were observed independently of their metabolic status and homeostasis model assessment–insulin resistance (HOMA-IR), leading to a conclusion that in PCOS, higher chemerin levels are independent from IR [47]. On the other hand, in a cross-sectional study carried on 100 PCOS cases and 70 controls attending for management of primary infertility, chemerin levels in the PCOS group were significantly positively correlated with fasting glucose levels, insulin levels, and HOMA-IR [43]. Moreover, the PCOS cases after three months of metformin therapy had significantly lower chemerin levels as compared with the nontreated PCOS cases [43]. Furthermore, in some recent studies already mentioned, the relationship between IR and PCOS has been studied [41,45]. Serum chemerin concentration in PCOS positively correlated with fasting insulin, fasting glucose, and HOMA-IR [41]. Serum chemerin levels were found significantly higher only in PCOS patients with T2D and impaired glucose tolerance compared with participants with normal glucose tolerance and controls [45]. No significant change was observed in the chemerin level between the control and PCOS groups without dysglycemia [45]. Moreover, the cutoff value of serum chemerin level to predict dysglycemia in PCOS individuals was very high (greater than 309.3 ng/mL), even if it could detect 79.8% of the PCOS individuals with T2D [45].

In conclusion, although the studies have not agreed unanimously, there seems to be a close relationship between chemerin levels and surrogate markers of IR or dysglycemia in women with PCOS. Chemerin could promote IR or worsen its severity, leading to impaired glucose metabolism (and T2D) in patients with PCOS.

### 3.3. Chemerin and Hyperandrogenism in PCOS

Hyperandrogenism is one of the diagnostic features for PCOS diagnosis and may lead to clinical manifestations such as hirsutism, acne, and alopecia [1,18,88,89]. Overproduction of androgens in PCOS arises from dysfunctional ovarian and adrenal steroidogenesis frequently worsened by progressive IR [90].

In a cross-sectional study including 106 patients with PCOS and 60 controls from Argentina, patients were classified as showing a hyperandrogenic (in presence of signs of biochemical and/or clinical hyperandrogenism) or normoandrogenic phenotype, and serum chemerin levels were assessed [47]. Serum chemerin levels were higher in PCOS patients compared with the control group. In PCOS patients, increased chemerin levels were found in both normoandrogenic and hyperandrogenic patients, thus independently from androgenic condition [47].

An experimental study evaluated hGCs and FF from patients undergoing IVF, confirming higher FF chemerin concentration and chemerin, CMKLR1, GPR1, and CCRL2 mRNA expression in hGCs in PCOS patients [46]. FF chemerin concentration was positively correlated with intrafollicular levels of testosterone (T) [46]. Furthermore, hGCs from non-PCOS women were collected and incubated with T for 24 h, showing that T upregulates the mRNA and protein levels of chemerin in a dose-dependent manner [46]. CCRL2 manifested a similar expression pattern, while CMKLR1 and GPR1 showed a tendency of decreasing, suggesting an adaptive downregulation following the increase in chemerin production [46]. These findings suggest that T might directly upregulate the expression of chemerin and its receptors in hGCs, probably amplifying the local impact of chemerin in the ovary [46].

The intrafollicular concentrations of androgens and adipokines were analyzed in a case–control study that compared PCOS patients, non-PCOS controls, and women with only PCOM on ultrasounds [48]. While higher FF chemerin levels in the PCOS group were confirmed, FF chemerin positively correlated with follicular levels of 17-OH-Pregnenolone, dehydroepiandrosterone, Δ4-androstenedione, and T [48]. Chemerin could be one of the possible mediators through which hyperandrogenism in PCOS induces alterations in oocyte maturation and perturbations in folliculogenesis [48].

In conclusion, although no relationship between biochemical hyperandrogenism and serum chemerin levels has been formally demonstrated, a direct effect of androgens on the ovarian production of chemerin has been detailed, suggesting that chemerin may act as a mediator of androgen effects on the ovary and ovulatory function. Unfortunately, experimental data on chemerin administration on the ovarian androgen pattern are lacking.

### 3.4. Chemerin and Ovarian Dysfunction in PCOS

PCOS is characterized by chronic anovulation, follicular arrest, and irregular cycles owing to ovarian dysfunction [91]. PCOS has multifaceted etiopathogenesis with genetic, metabolic, hormonal, and environmental contributions [88]. The primary underlying mechanism for PCOS seems to constitute an aberrant signaling in the hypothalamic–pituitary–ovarian (HPO) axis, which leads to hormonal secretion rhythms that deviate from normal as well as abnormal follicular development dynamics [91]. On the other hand, even though neuroendocrine regulation in PCOS shows robust differences, ranging from GnRH pulsatility to hormonal feedback and neuropeptide signaling, further modulatory effects conferred by metabolic dysregulation (e.g., insulin and leptin resistance and hyperandrogenism) indirectly define potential physiopathologically relevant causes [92]. As previously detailed in the studies focused on the effects of hyperinsulinism or hyperandrogenism on FF, chemerin may act as an insulin or androgen mediator and may have an autocrine/paracrine effect in the ovarian environment, impairing ovary functioning. Indeed, insulin-induced chemerin expression in FF may amplify intraovarian IR [44]. At the same time, chemerin may be a mediator of androgens effects on ovarian function [46,48]. Other studies have added further contributions to the knowledge of chemerin’s effects on ovarian function.

In a study involving pregnant rats hyperandrogenized with T, female offspring manifested irregular ovulatory phenotypes or anovulatory phenotypes, and adipokine mRNA expression in the ovary was measured [49]. Chemerin mRNA and protein levels were altered only in the irregular ovulatory phenotype, showing higher levels than in the anovulatory phenotype and control groups. Therefore, chemerin may contribute to the ovarian alterations observed in the irregular ovulatory phenotype, including low levels of estradiol and progesterone, and high levels of T [49]. In the same experimental model, gonadal adipose tissue was analyzed, finding decreased expression of chemerin only in the irregular ovulatory subjects [55]. These results were attributed by the authors to increased levels of lipogenesis of this specific phenotype compared with the anovulatory phenotype [55]. Indeed, peroxisome proliferator-activated receptor-ɣ, a ligand-activated transcription factor that promotes lipid production and storage through the lipogenesis pathway, seems to be able to determine a decrease in chemerin expression [93].

To evaluate the effects of chemerin on ovarian steroidogenesis, an experimental study analyzed FF and luteinized hGCs from normal-weight PCOS and non-PCOS patients undergoing IVF and the effects of chemerin on human ovarian granulosa-like tumor cell line (KGN) [52]. The results confirmed higher concentrations of chemerin within the FF in PCOS patients and a higher expression of the chemerin gene, *RARRES2*, in luteinized hGCs recovered from PCOS women [52]. Significant overexpression of CMKLR1 in luteinized hGCs was found [52]. Moreover, chemerin decreased progesterone secretion by the KGN. This inhibition of the progesterone secretion was abolished in a dose-dependent manner in response to a nanobody raised against CMKLR1. Progesterone secretion inhibition was higher in the PCOS group, with a decrease of about 75% [52]. The progesterone secretion inhibition was associated with a decrease in *STAR* mRNA expression that was reverted by incubation with the nanobody against CMKLR1 [52].

Many studies on rat models of PCOS have analyzed the role of chemerin on follicular growth, development, and function in PCOS, highlighting its capacity to induce low-grade inflammation [53,94]. High levels of chemerin, as a ligand for CMKLR1-expressing monocytes in the blood, provoked local ovarian inflammation, leading to granulosa cell apoptosis, follicular growth arrest, and anovulatory infertility [94]. In a second study, the expression of chemerin and CMKLR1 were significantly increased in PCOS ovarian tissues compared with that of healthy controls [53]. Furthermore, chemerin promoted the expression of light chain 3-II protein, indicating promotion of autophagy [53]. Chemerin and CMKLR1 promoted autophagy through the inhibition of the phosphoinositide 3-kinase (PI3K)/Akt/mammalian target of rapamycin and mitogen-activated protein kinase pathways [53]. Furthermore, they promoted the expression of the autophagy inductor Un-51-like autophagy-activating kinase 1 [53].

In summary, available studies have shown how high levels of chemerin, acting on CMKLR1, impair normal ovarian function in PCOS and may be a potential effector for many pathophysiological mechanisms of this disease. In women with PCOS, chemerin may be responsible for progesterone secretion disturbances [52] and for ovarian inflammation and autophagy [53,94]. Even if the relationship between reduced progesterone secretion and enhanced inflammation/autophagy needs to be elucidated, these effects may be associated with PCOS pathogenesis.

### 3.5. Chemerin in Infertile Patients with PCOS

The primary clinical presentation of PCOS, characterized by chronic anovulatory symptoms and hormonal imbalances, usually results in menstrual cycle disturbances that decrease natural conception rates [9]. Fertility management in PCOS needs a multipronged approach with lifestyle modifications, pharmacological interventions, and, when indicated, IVF treatment [95].

As detailed in the previous section, many studies have aimed to analyze the influence of chemerin on ovulatory function. On the other hand, few and sparse data regarding the effects of chemerin on fertility outcome in infertile patients with PCOS are available. In a case–control study involving 30 nonobese PCOS patients and 23 nonobese controls, patients were further divided into two groups in relation to levels of intrafollicular chemerin, and their IVF outcomes were studied [46]. The high-FF-chemerin group had a lower oocyte recovery rate and lower high-quality embryo rate than the low-FF-chemerin group, suggesting an adverse effect of chemerin on IVF outcomes [46]. In a further study involving a small number of infertile lean PCOS and non-PCOS women who underwent IVF, chemerin levels in serum and FF were higher in subjects who did not achieve a clinical pregnancy [51]. Cutoff values of 36.2 ng/mL in the FF chemerin level and of 12.7 ng/mL in the serum chemerin level were found to predict clinical pregnancy [51].

Therefore, although data available are limited, the presence of high levels of chemerin appears to be an unfavorable prognostic factor for IVF outcomes in infertile women with PCOS.

### 3.6. Chemerin and Pregnancy Complications: Potential Role in PCOS

PCOS is being progressively recognized as a condition with systemic consequences in pregnancy status. Women with PCOS are at a high risk of pregnancy and obstetric complications, including GDM, hypertensive disorders of pregnancy (such as PE), preterm delivery, and miscarriage [10,96]. Increased risk of GDM in PCOS is closely linked with underlying insulin resistance and hyperinsulinemia, which are characteristic features of the syndrome, even in lean women [97]. In addition to the increased risk of cardiovascular disease, women with PCOS also more frequently experience PE, a significant complication of new-onset hypertension and proteinuria during pregnancy [98] that may also be mediated by a combination of factors, including chronic inflammation, endothelial dysfunctions, and abnormal placentation [99]. In addition, there is increased risk of preterm birth, cesarean section, and adverse neonatal outcomes such as low birth weight and admission to neonatal intensive care units [100].

#### 3.6.1. Chemerin and GDM

GDM is a metabolic disease with significant adverse maternal and fetal consequences for which women with PCOS are at increased risk. IR and hyperinsulinemia, which are intrinsically associated with PCOS, help explain this higher risk, influencing glucose metabolism during pregnancy [101] and with increased prevalence of its persistence [102].

Chemerin’s role during physiological pregnancy has not yet been elucidated. The association between chemerin levels and GDM has long been studied, with sometimes conflicting results. In 2021, a meta-analysis of nine studies conducted from 2012 to 2018, including 772 GDM patients and 857 controls, concluded that circulating chemerin levels in women with GDM did not differ significantly from those of controls with normal glucose tolerance [55].

This finding was the result of conflicting data among the various studies, which have continued to be reported in subsequent years. Some case–control studies have not found significant differences in chemerin levels between women with and without GDM [56,63,64,65,67]. Most studies in the literature, however, have reached opposite conclusions, proposing also to use the levels of this adipokine as a marker of GDM [58,60,61,62,68,69,71]. At the same time, a study found serum chemerin in the second trimester of pregnancy inversely associated with GDM (OR 0.85) [64]. Participants with chemerin < 8.03 ng/mL presented a 3.6-fold higher risk of GDM compared with participants with > 10.2 ng/mL [64]. These results had previously been achieved also in a Danish cohort [103]. A case–control study analyzed adipokines in the saliva of pregnant women and discovered a significant association of salivary chemerin with GDM diagnosis [61]. A subsequent study reached opposite results, not finding differences in salivary chemerin between GDM and no-GDM groups [67]. In a Chinese cohort, two genetic variants in the *RARRES2* gene (rs4721 and rs17173608) were associated with plasma levels of chemerin and HOMA-IR and offered protection against the development of GDM in Chinese women [69].

The potential mechanisms through which chemerin could act to influence GDM development and its associated obstetric complications have also been studied. In GDM, chemerin induced placental inflammation through the recruitment of macrophage cells and release of interleukin (IL)-18 and IL-1β [70]. This process reduced placenta-derived exosomal miR-140-3p and miR-574-3p expression, promoting the proliferation, migration, and tube formation of umbilical vein endothelial cells [70]. Another study attributed chemerin to a protective mechanism, as it seemed to reduce placenta mitochondrial dysfunction in GDM patients, promoting the expression of disulfide-bond A oxidoreductase-like protein and inhibiting the cyclic GMP-AMP synthase stimulator of interferon genes pathway [72].

Data on colostrum and human milk in GDM women revealed that chemerin is significantly increased and positively correlated with blood chemerin levels [58,68]. Colostrum and breast milk chemerin levels showed an independent association with infant weight at 6 weeks postpartum, suggesting a possible role in contributing to childhood obesity [68].

Therefore, evidence on the association between GDM and chemerin is contradictory. Such discrepancies may be attributed to different gestational phases of pregnancy, time of blood collection, the ethnicity of the study population, the genetic makeup of subjects, and the sample numbers of these studies. Even if there are no studies in the literature on chemerin in GDM in patients with PCOS, it is possible to speculate that chemerin also plays an etiological role in the pathogenesis of GDM in these patients.

#### 3.6.2. Chemerin and PE

As already reported, women with PCOS have an increased risk for PE, a potentially serious pregnancy complication. PE is defined as hypertension after 20 weeks gestation in association with at least one other target organ manifestation (including proteinuria, renal insufficiency, abnormal liver function, thrombocytopenia, and uteroplacental insufficiency) [104]. The metabolic disturbances and chronic inflammation in PCOS, such as IR and high androgen levels, could be responsible for PE development [105]. Thus, PE is more common in women with PCOS, and early identification and management of these risk factors during pregnancy are crucial to minimize the occurrence and severity of this serious complication [10].

Two recent meta-analyses agreed that women with PE exhibited a statistically significant elevation in serum chemerin concentration when compared with control subjects [73,74]. In patients with PE, circulating chemerin levels were elevated even when measured before or after PE diagnosis (before PE diagnosis: SMD 1.41, 95% CI 1.00 to 1.83; after PE diagnosis: SMD 1.40, 95% CI 0.96 to 1.84) [74]. These results suggested a possible role for chemerin as a biomarker for future predictive models of PE [74]. Severe PE was associated with a more remarkable increment of serum chemerin as compared with mild PE (SMD 67.89 ng/mL; 95% CI 25.64 to 110.14 ng/mL) [73]. The mean BMI of the pregnant women may modify the difference in circulating chemerin levels between women with and without PE [73]. Recent studies confirmed a significant positive correlation between PE and chemerin levels [75,76,78,80]. Circulating chemerin levels were found to be significantly elevated in early-onset PE, established PE, and in patients preceding the diagnosis of PE, compared with healthy controls [80].

Experimental studies better defined the pathophysiological mechanisms linking PE to chemerin. In a study involving an in vivo PE rat model, chemerin aggravated PE, while the inhibition of CMKLR1 suppressed M1 macrophage polarization and alleviated PE [77]. Moreover, chemerin inhibited many mechanisms involved in PE etiology, including macrophage-induced trophoblast invasion and migration and macrophage-mediated angiogenesis [77]. In mice, exogenous chemerin induced a PE-like syndrome involving hypertension, proteinuria, endotheliosis, diminished trophoblast invasion, and a disorganized labyrinth layer [78]. Chemerin upregulated the soluble Fms-like tyrosine kinase-1 (sFlt-1), a biomarker of PE, and the inflammation markers nuclear factor-κB, tumor necrosis factor-α, and IL-1β [78]. Overexpression of placental chemerin production also disrupted trophoblast lipid metabolism, increasing circulating and placental levels of cholesterol, serum levels of lysophosphatidic acid, HDL-C, and LDL-C [79]. Chemerin overexpression increased the production of lysophospholipids and phospholipids [79]. These mechanisms induce vascular damage and trophoblastic invasion and contribute to the development of dyslipidemia in PE.

Circulating chemerin levels correlated positively with sFlt-1/placental growth factor ratio, an indicator of PE severity [78]. Chemerin overexpression in human trophoblasts upregulated sFlt-1 and reduced vascular endothelial factor-A [78]. Chemerin inhibited migration, invasion, and tube formation during coculture with human umbilical vein endothelial cells [78]. CMKLR1 inhibition prevented the latter phenomenon, although it did not reverse the chemerin-induced downregulation of the PI3K/Akt pathway [78].

In a prospective cohort study of 467 women with singleton pregnancies, preeclamptic placentas released more chemerin than those of healthy controls [76]. Chemerin constricted both healthy and preeclamptic chorionic plate arteries, while the CMKLR1 receptor antagonist α-norethisterone acetate blocked these constrictor effects [76]. Moreover, placental perfusion with pravastatin and fluvastatin suppressed the release of chemerin from perfused healthy and preeclamptic placentas, thus also reducing chemerin-induced constrictor effects. Therefore, this study proposed considering statins as a potential therapeutic strategy to prevent or treat PE at an early stage [76].

Finally, a cohort study found that serum chemerin measured at ≈35 gestational weeks positively correlated with the occurrence of postpartum hypertension in patients with PE (OR 4.01 for BP ≥ 130/80 mmHg; OR 1.70 for BP ≥ 140/90 mmHg) [75].

In conclusion, the literature seems to agree that there is a close link between serum chemerin concentration and risk of PE. Chemerin appears to play a role in mechanisms that promote and/or aggravate PE. Increased chemerin levels before PE or in early PE stages could make it a possible PE marker [80]. As for GDM, there are no studies on PE in patients with PCOS. However, in the PCOS patient, because of the alterations of trophoblastic and placental invasion evidenced as the pathogenic basis of PE, it is possible to hypothesize the role of chemerin as an additional effector of damage [10].

## 4. Discussion

The current study is the first comprehensive review aimed to provide evidence about the critical association between chemerin, PCOS, and PCOS-related comorbidities and complications. Many data from recent meta-analyses and experimental clinical studies on animal and human models were intercepted and discussed according to specific targets.

**Figure 1:**
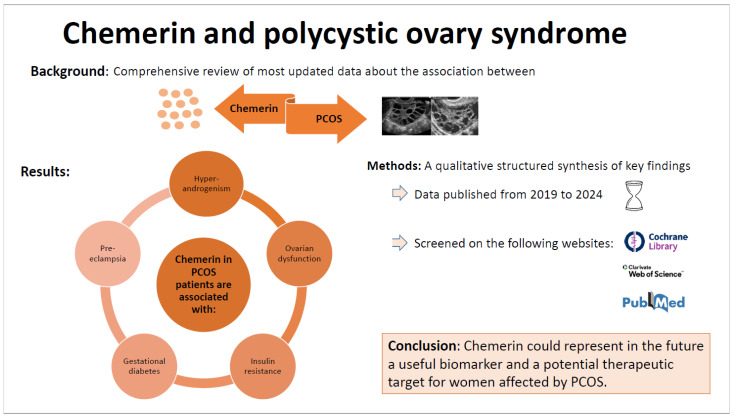
Representation of the literature analysis and main findings about the relationship between chemerin and PCOS, and its related comorbidities/complications.

Our findings reveal that chemerin seems to be involved in several PCOS characteristics, such as ovarian dysfunction [52,53,94], hyperandrogenism [48], and reproductive outcomes [51], but also correlates with metabolic disturbances related to PCOS, such as IR [44] and obesity [41]. Figure 1 provides a graphical representation of our literature analysis and findings. In addition, even if the available studies on this association do not contain subgroup analyses on the presence of pregestational PCOS (Table 5) [55,73,74], it may play a role in the increased risk of pregnancy complications in women with PCOS (GDM and PE) [10].

Several meta-analyses (see Table 7) have consolidated the association between PCOS and chemerin. Notwithstanding that different sample sizes across meta-analyses could be a limitation to the comparison among their results, serum chemerin levels have been shown to be higher in women with PCOS than in controls. A landmark meta-analysis of 22 studies [36] demonstrated that serum and FF chemerin concentrations, and ovarian chemerin mRNA expression, were higher in patients with PCOS than in controls. The increase in circulating chemerin levels was observed in subgroup analyses, regardless of BMI groups or sample size [36]. Other meta-analyses [20,37] confirmed these findings, even if data variability between overweight/obese (SMD 2.62, 95%CI 1.78–3.46) and nonobese (SMD 1.31, 95%CI 0.62–1.99) populations was observed [37]. A more recent meta-analysis [38] reinforced the consensus and reported higher levels of circulating chemerin in PCOS, thus reiterating its potential as a biomarker in PCOS. Moreover, the exact contribution of chemerin to PCOS pathology and its role in nonobese populations remains inconsistent.

Chemerin acts on its receptors (CMKLR1, CCRL2, GPR1) in the HPO axis and peripheral tissues [46]. Chemerin seems to affect hypothalamic GnRH expression and serotonin synthesis, possibly influencing appetite and reproductive hormone release [106,107]. Locally, chemerin acts at the ovarian level when it inhibits IGF-1-induced steroidogenesis and FSH activity, while also downregulating its main steroidogenic enzymes, further determining impairment of both folliculogenesis and steroidogenesis [108,109]. Such mechanisms add a great deal to the hormonal imbalances and metabolic dysfunctions of PCOS, laying a solid foundation for the possible role of chemerin in its pathophysiology.

The present review has strengths and limitations. First, the current is the first comprehensive review aimed to analyze the interactions between chemerin and PCOS and their potential pathophysiological links and clinical significance. Second, we included only studies published during the last five years. Third, we included experimental and clinical studies on the animal and human models of PCOS. However, there are also notable limitations. The current review included studies with different sample sizes. Specifically, we did not exclude studies with small sample sizes that might be underpowered to detect significant differences in chemerin effect. Longitudinal studies on the association of serum chemerin levels with the course of PCOS also need to be well validated. This makes comparisons difficult, particularly regarding control group characterization and PCOS diagnosis and phenotype stratification. There remain, however, important limitations to generalizability, as differing methodological quality across many studies resulted in these having low or heterogeneous samples. Limits also include the absence of standardized procedures or removal of confounding factors in chemerin dosing, considering, for example, the fluctuations of this adipokine in relation to meals [39]. These drawbacks emphasize the need for improved controlled and uniform research designs.

In order to provide more insights into the function of chemerin in PCOS, some crucial directions for further research should be focused on. Longitudinal studies are required because, at the moment, it is not possible to ascertain whether high chemerin levels are causative for PCOS or merely a consequence of the metabolic and hormonal features associated with the syndrome. Clarifying this relationship would provide insight into the pathophysiology of PCOS. In addition, consensus protocols are needed for the measurement of individual chemerin levels according to PCOS phenotype characteristics. This would indicate consistency across studies, enabling comparison of findings. The other pivotal aspect that can be investigated is the evaluation and development of therapeutics that directly target chemerin pathways. They might provide new therapeutic approaches for the management of metabolic factors and reproductive disorders, contributing to PCOS pathology alongside long-term prevention of associated disorders. Finally, a deeper study of the role of chemerin in pregnancy outcomes is needed. Indeed, knowing its role in maternal and fetal health, specifically in diseases such as GDM and PE, may result in better screening procedures and more successful treatment plans for mothers with PCOS during pregnancy. Moving forward, much progress could be made by meeting these priorities in advancing the management of PCOS.

## 5. Conclusions

Our comprehensive review outlines the emerging evidence supporting chemerin as a key player of general relevance to the pathogenesis of PCOS and PCOS-related comorbidities and complications (Figure 1). Therefore, it underlines that chemerin could be a potential biomarker and therapeutic target for this multifaceted syndrome in the future. However, the current review has many limitations, including the presence of underpowered studies with small sample sizes and different methodological quality, a lack of longitudinal studies, and an absence of standardized procedures in chemerin dosing. Thus, we need better longitudinal and high-quality studies, pathophysiological insights, and diagnostic protocols to inform clinical decision-making beyond the significant scientific findings. Filling these gaps will help further define the intricate interplay among chemerin, the HPO axis, and PCOS comorbidities. In the end, all of this may lead to new therapies helping women suffering from this complex syndrome to have a better quality of life.

## Figures and Tables

**Table 1:** Main clinical studies on potential correlations between chemerin and obesity in women with PCOS.

Reference	Country	Population	Study Design	Sample Size and Groups	Study Aim	Main Findings
Martìnez-Garcia et al. (2019) [39]	Spain	Humans	Prospective cross-sectional study	53 [17 PCOS (9 nonobese/8 obese) vs. 17 healthy female controls without clinical and/or biochemical hyperandrogenism (9 nonobese/8 obese) vs. 19 healthy male controls (10 nonobese/9 obese)]	To study the influence of sex hormone imbalances and obesity on the circulating levels of a panel of metabolic markers—including chemerin—in the fasting state and 120 min after single oral loads of glucose, lipids, and protein (300 kcal each)	No association between serum chemerin levels and PCOS (*p* = 0.240). Chemerin was significantly associated with obesity (*p* = 0.015). In both groups, chemerin significantly decreased after glucose (*p* < 0.001) and lipid challenges (*p* < 0.001).
Bongrani et al. (2019) [40]	France	Humans	Prospective cross-sectional study	78 women [23 PCOS (13 normal weight/10 obese) vs. 28 with only PCOM (13 normal weight/15 obese) vs. 27 controls (12 normal weight/15 obese)]	To investigate FF adipokine concentration and GC mRNA expression in PCOS women in comparison with controls and women with only PCOM	FF chemerin concentration was significantly associated with obesity (*p* < 0.001). In normal-weight subjects, chemerin was associated with PCOS compared with BMI-matched PCOM and controls (*p* < 0.001). GC mRNA chemerin levels were higher in the obese PCOS group (*p* < 0.001).
Niepsuj et al. (2024) [41]	Poland	Humans	Retrospective cross-sectional study	116 women [88 PCOS (47 with BMI < 25.0/18 with 25.0 < BMI < 30.0/23 with BMI > 30.0) vs. 28 healthy controls]	To investigate the effect of exposure to tobacco smoke and abnormal body weight on selected circulating peptide hormones—including chemerin—and their association with metabolic and hormonal parameters in PCOS women	Chemerin was not associated with PCOS (*P NS)*. Among PCOS women, serum chemerin levels were not associated with tobacco smoke exposure but positively correlated with BMI (r = 0.23; *p* < 0.035) and WHR values (r = 0.26; *p* < 0.017).
Abdullateef et al. (2024) [42]	Iraq	Humans	Prospective cross-sectional study	88 women [32 healthy controls (16 normal weight/16 overweight-obese) vs. 56 PCOS (26 normal weight/30 overweight-obese)]	To compare serum levels of irisin, chemerin, and insulin in women with PCOS and controls with respect to body weight and BMI	Serum chemerin was significantly higher in PCOS (*p* < 0.001). This association was not maintained between normal-weight PCOS and BMI-matched controls (*p* = 0.071), but only among overweight and obese PCOS and BMI-matched controls (*p* < 0.001).

BMI: body mass index; FF: follicular fluid; GC: granulosa cells; mRNA: messenger ribonucleic acid; NS: not significant; PCOM: polycystic ovarian morphology; PCOS: polycystic ovary syndrome; WHR: waist–hip ratio.

**Table 2:** Main clinical studies on potential correlations between chemerin and IR in women with PCOS.

Reference	Country	Population	Study Design	Sample Size and Groups	Study Aim	Main Findings
Foda et al. (2019) [43]	Egypt	Humans	Prospective cross-sectional study	170 women between 21 and 26 years [100 PCOS (50 obese/50 normal weight) studied before and after three months of metformin therapy vs. 70 healthy controls (35 obese/35 normal weight) with only basal evaluation]	To compare serum chemerin with PCOS diagnosis and correlate them with IR parameters and with hormonal profile, and to study the effects of three months of metformin therapy on serum chemerin in obese and normal-weight PCOS cases	Serum chemerin levels were significantly higher in obese (*p* < 0.0001) and normal-weight (*p* < 0.0001) PCOS cases as compared with the BMI-matched controls. Chemerin was correlated with glucose levels (*p* < 0.0001), insulin levels (*p* < 0.0001), and HOMA-IR (*p* < 0.002) in obese PCOS cases. Serum chemerin correlated with glucose levels (*p* < 0.016), insulin levels (*p* < 0.002), and HOMA-IR (*p* < 0.001) also in normal-weight PCOS cases.
Li et al. (2019) [44]	China	Humans	Prospective cross-sectional study	101 women [50 non-PCOS (26 without IR/24 with IR) vs. 51 PCOS (25 without IR/26 with IR)]	To investigate the relationship between IR, defined as fasting insulin ≥ 15 mIU/mL or HOMA-IR ≥ 2.68, and hlGCs and FF chemerin levels in PCOS patients	FF chemerin levels were significantly elevated in PCOS with IR compared with PCOS without IR (*p* < 0.05), controls with IR (*p* < 0.05), and controls without IR (*p* < 0.05). hlGC chemerin mRNA expression was significantly higher in PCOS with IR compared with controls without IR (*p* < 0.01); hlGC chemerin levels were significantly higher in PCOS with IR compared with controls with IR (*p* < 0.001). Insulin induced the expression of chemerin in hlGCs.
Bose et al. (2024) [45]	India	Humans	Prospective cross-sectional study	126 women [93 PCOS (34 NGT/33 IGT/26 T2D) vs. 33 healthy controls]	To study the role of serum chemerin in identifying dysglycemic PCOS	Serum chemerin levels were significantly higher in PCOS than in controls (*p* < 0.05). In the subgroup analysis, serum chemerin was higher in T2D PCOS than in healthy controls (*p* < 0.0001) and NGT PCOS (*p* < 0.001); serum chemerin was also higher in IGT PCOS than in healthy controls (*p* < 0.001) and NGT PCOS (*p* < 0.01). Serum chemerin was significantly associated with HOMA-2β (r = −0.5326, *p* = 0.0004) but no association was observed with HOMA-IR (r = 0.3499, *p* = 0.0798).

BMI: body mass index; hlGCs: human luteinized granulosa cells; HOMA-2β: homeostatic model 2 assessment of β-cell function; HOMA-IR: homeostasis model assessment-insulin resistance; FF: follicular fluid; IGT: impaired glucose tolerance; IR: insulin resistance; NGT: normal glucose tolerance; PCOS: polycystic ovary syndrome; T2D: type 2 diabetes mellitus.

**Table 3:** Main experimental and clinical studies on potential correlations between chemerin and hyperandrogenism in PCOS.

Reference	Country	Population	Study Design	Sample Size and Groups	Study Aim	Main Findings
Wang et al. (2019) [46]	China	Humans	Prospective case–control study	53 women with BMI < 25.0 (30 PCOS vs. 23 non-PCOS)	To assess the level of FF chemerin and its receptors from lean patients with and without PCOS, to evaluate association between ovarian hyperandrogenism and chemerin expression, and to examine the relationship between chemerin and IVF outcomes	In PCOS were found significantly higher: FF chemerin levels (*p* < 0.01); serum chemerin levels (*p* < 0.05), CMKLR1 (*p* < 0.01), CCRL2 (*p* < 0.01), and GPR1 (*p* < 0.01) mRNA levels in GC. FF chemerin significantly correlated with FF testosterone levels (r = 0.3566, *p* < 0.01) and FF LH levels (r = 0.274, *p* < 0.05). GC incubation with testosterone significantly increased chemerin mRNA (*p* < 0.05), CMKLR1 mRNA (*p* < 0.05), and GPR1 mRNA (*p* < 0.05). Higher FF chemerin levels were associated with lower oocyte recovery rates (percentage of embryos transferred and frozen per oocyte) (*p* < 0.05) and lower percentages of high-quality embryos in IVF (*p* < 0.01).
Abruzzese et al. (2020) [47]	Argentina	Humans	Prospective case–control study	166 women between 18 and 38 years old (106 PCOS vs. 60 healthy controls)	To study whether serum chemerin levels are related to the presence of hyperandrogenic phenotype (presence of biochemical and/or clinical hyperandrogenism)	Serum chemerin levels were significantly higher in PCOS (*p* < 0.0001, adjusted for age and BMI), independently from normoandrogenic or hyperandrogenic phenotype, compared with controls.
Bongrani et al. (2022) [48]	France	Humans	Prospective cross-sectional study	106 women between 21 and 42 years old (31.6 ± 4.7) (30 PCOS (15 normal weight/15 obese) vs. 29 with only PCOM (15 normal weight/14 obese) vs. 47 healthy controls (26 normal weight/21 obese)	To characterize the expression profile of the androgens in FF and its relationship with the FF concentration of adipokines	FF chemerin was significantly higher in normal-weight PCOS group than in BMI-matched control (*p* < 0.0001) and PCOM groups (*p* < 0.05); FF chemerin was also significantly higher in obese PCOS than in BMI-matched control (*p* < 0.01) and PCOM groups (*p* < 0.01). In FF, chemerin levels correlated with FF Δ4-androstenedione (r^2^ = 0.09, *p* < 0.01), DHEA (r^2^ = 0.11, *p* < 0.001), 17OH-pregnenolone (r^2^ = 0.05, *p* < 0.05), and testosterone (r^2^ = 0.07, *p* < 0.01) levels.

BMI: body mass index; CCRL2: C-C motif chemokine receptor-like 2; CMKLR1: chemokine receptor-like 1; DHEA: dehydroepiandrosterone; FF: follicular fluid; GC: granulosa cells; GPR1: G protein-coupled receptor 1; IVF: in vitro fertilization; LH: luteinizing hormone; mRNA: messenger ribonucleic acid; PCOM: polycystic ovarian morphology; PCOS: polycystic ovary syndrome.

**Table 4:** Main experimental and clinical studies on potential correlations between chemerin and ovarian dysfunction in PCOS.

Reference	Country	Population	Study Design	Sample Size and Groups	Study Aim	Main Findings
Abruzzese et al. (2019) [49]	Argentina	Rats	Prospective cross-sectional study	194 female rats [88 prenatally hyperandrogenized (39 with irregular ovulatory phenotype/37 with anovulatory phenotype/12 with regular ovulatory phenotype) vs. 106 healthy controls]	To evaluate the effect of prenatal hyperandrogenization as experimental model of PCOS on ovarian steroidogenesis and adipokine levels	In the irregular ovulatory phenotype subgroup, ovarian mRNA expression of chemerin and chemerin protein levels were significantly higher compared with controls (*p* < 0.05) and anovulatory subgroup (*p* < 0.05).
Huang et al. (2020) [50]	China	Humans	Prospective case–control study	24 (12 PCOS vs. 12 healthy controls) (PCOS subgroup)	To develop and validate an LC/MRM-MS-based targeted proteomic method with immunoaffinity precipitation for the enrichment and detection of low abundance chemerin isoforms in human biofluids	Total chemerin resulted increased in PCOS (serum, *p* < 0.001; FF, *p* < 0.01), but only 156F (serum, *p* < 0.001; FF, *p* < 0.01) and 157S isoforms (serum, *p* < 0.01; FF, *p* < 0.01) were increased. Chemerin isoforms may be suitable biomarkers for PCOS.
Kabil Kucur et al. (2021) [51]	Turkey	Humans	Prospective case–control study	78 nonobese women (33 PCOS vs. 43 controls)	To evaluate the predictive value of serum and FF chemerin levels on IVF outcome and clinical pregnancy rate in lean patients with PCOS	PCOS was associated with higher serum and FF chemerin levels (*p* < 0.01). Serum and FF chemerin levels were higher in subjects without clinical pregnancy (*p* < 0.05).
Estienne et al. (2021) [52]	France	Humans	Prospective case–control study	43 normal-weight women (17 PCOS vs. 26 healthy controls)	To investigate progesterone production and chemerin system expression in hGCs and to study the effects of exogenous 200 ng/mL chemerin on progesterone secretion in KGN	Higher concentration of chemerin in FF (*p* < 0.0001), expression of the *RARRES2* gene in hGCs (*p* < 0.01) and expression of *CMKLR1* (*p* < 0.0001) in PCOS. Chemerin decreased progesterone secretion by KGN cells (*p* < 0.001); this inhibition was abolished in response to a nanobody against CMKLR1 (*p* < 0.001).
Luo et al. (2021) [53]	China	Rats	Prospective cross-sectional study	36 rats [10 high-fat diet rats vs. 10 testosterone propionate-treated rats vs. 6 high-fat diet and testosterone propionate-treated rats (PCOS model) vs. 10 controls]	To investigate chemerin and CMKLR1 levels in a rat model of PCOS and the effects in vitro of ectopic chemerin on autophagy mechanisms in hGCs derived from a solid primary granulosa tumor	Chemerin (*p* < 0.001) and CMKLR1 (*p* < 0.001) were overexpressed in PCOS model rats compared with controls. Ectopic chemerin promoted autophagy in hGCs in vitro through inhibiting the PI3K/Akt/mTOR pathway (*p* < 0.001).
Ferrer et al. (2023) [54]	Argentina	Rats	Prospective cross-sectional study	15 female rats [10 prenatally hyperandrogenized (5 with irregular ovulatory phenotype/5 with anovulatory phenotype) vs. 5 healthy controls]	To investigate in prenatally hyperandrogenized rats, as experimental model of PCOS, adipokine expression in gonadal adipose tissue	The irregular ovulatory phenotype rats presented levels of chemerin in gonadal adipose tissue significantly lower compared with controls and anovulatory phenotype rats (*p* < 0.05).

ART: assisted reproductive technology; CMKLR1: chemokine receptor-like 1; hGCs: human granulosa cells; FF: follicular fluid; IVF: in vitro fertilization; KGN: human granulosa-like tumor cell line; LC/MRM-MS: liquid chromatography/multiple reaction monitoring-mass spectrometry; mTOR: mammalian target of rapamycin; PCOS: polycystic ovary syndrome; PE: preeclampsia; PI3K: phosphoinositide 3-kinase; RARRES2: retinoic acid receptor responder 2.

**Table 5:** Main experimental and clinical studies on potential correlations between chemerin and pregnancy complications.

Reference	Country	Population	Study Design	Sample Size and Groups	Study Aim	Main Findings
Chemerin and GDM						
Sun et al. (2021) [55]	China	Humans	Meta-analysis	2981 pregnant women (1493 with GDM vs. 1488 controls)	To systematically evaluate the correlations between the serum levels of adipokines and GDM	No significant differences in circulating chemerin levels in women with GDM (SMD 0.77, 95%CI −0.07 to 1.61, *p* = 0.07).
Ueland et al. (2019) [56]	Norway	Humans	Prospective cohort study	273 pregnant women	To evaluate if circulating adipokines and monocyte/macrophage markers (measured at 14–16, 22–24, 30–32, 36–38 weeks of pregnancy and 5 years postpartum) were dysregulated in patients developing GDM	No correlations between serum levels of chemerin and GDM during pregnancy and 5 years postpartum, unadjusted and adjusted for BMI, age, CRP, diabetes in family, and parity.
Huang et al. (2019) [57]	China	Humans and mice	Prospective case–control study	16 pregnant women (8 with GDM vs. 8 without GDM) and 32 pregnant mice (chow-fed group vs. high-fat-diet-fed group)	To investigate chemerin and GPR1 expression in the placenta and their correlation with carbohydrate homeostasis during pregnancy in humans and mice	RT-PCR analysis showed increased chemerin (*p* < 0.05) and decreased GPR1 (*p* < 0.05) in GDM patients’ placentas. In high-fat-diet-fed pregnant mice were found higher levels of serum chemerin (*p* < 0.05) and lower levels of GPR1 mRNA expression (*p* < 0.01).
Ustebay et al. (2019) [58]	Turkey	Humans	Prospective case–control study	53 pregnant women (26 with GDM vs. 27 without GDM)	To determine the concentrations of chemerin and dermcidin in the postpartum period in the milk and blood of mothers with and without GDM	Increased concentrations of chemerin in the postpartum period in milk (*p* < 0.05) and blood (*p* < 0.05) in patients with GDM.
Aviram et al. (2020) [59]	Israel	Humans	Prospective observational study	75 pregnant patients with GDM (26 subjects requiring medications vs. 49 not requiring medications)	To evaluate the relationship between adipokines and glycemic control in GDM	Chemerin levels were not significantly higher in the group who required medications (*p* = 0.10).
Francis et al. (2020) [60]	United States	Humans	Prospective case–control study	321 pregnant women (107 with GDM vs. 214 without GDM matched on a 1:2 ratio based on maternal age, ethnicity and gestational week of blood collection)	To investigate the association of a panel of serum adipokines at 10–14, 15–26, 23–31, and 33–39 gestational weeks in women with and without GDM	Serum chemerin concentrations higher in GDM cases (10–14 weeks, *p* 0.05; 15–26 weeks, *p* < 0.001; 23–31 weeks, *p* < 0.05; 33–39 weeks, *p* < 0.05). After adjustment for maternal age, gestational week, parity, and family history of diabetes, serum chemerin at 10–14 gestational weeks was associated with GDM risk (*p* for linear trend < 0.01).
Okten and Bildacı (2020) [61]	Turkey	Humans	Prospective case–control study	91 pregnant women (18 with GDM vs. 73 without GDM)	To find a possible new and tolerable screening technique for GDM using salivary levels of leptin and chemerin	Chemerin was quantifiable in salivary samples. Salivary chemerin concentrations were significantly higher in patients with GDM (*p* < 0.001).
Schuitemaker et al. (2020) [62]	Netherlands	Humans	Retrospective case–control study	150 pregnant women (50 with GDM vs. 100 without GDM matched on a 1:2 ratio based on gestational age, maternal age and BMI)	To evaluate whether sFRP4 concentration in the first trimester of pregnancy was individually, or in combination with adipokines, associated with the development of GDM	Serum chemerin significantly increased in GDM (*p* < 0.05). In a univariate logistic regression analysis of logarithmic transformed data, chemerin showed significant OR (6.51, 95%CI 1.61–26.4, *p* = 0.027; AUC 0.601, 95%CI 0.503–0.700). In the first trimester of pregnancy, a multimarker model including sFRP4, leptin, chemerin, and adiponectin was associated with the development of GDM (AUC 0.699, 95%CI 0.605–0.793, *p* = 0.024).
Ebert et al. (2020) [63]	Germany	Humans	Prospective cross-sectional study	222 women [148 pregnant (74 with GDM/74 without GDM) vs. 74 nonpregnant healthy women]	To determine whether seven circulating adipokines were associated with GDM or were altered by metabolic and weight changes during pregnancy itself	Serum levels of chemerin significantly higher in women with GDM and pregnant controls than in nonpregnant controls (*p* < 0.001). Serum chemerin was not associated with GDM in pregnant patients (*p* = 0.192).
Mosavat et al. (2021) [64]	Malaysia	Humans	Prospective case–control study	96 pregnant women between 18 and 45 years (53 with GDM vs. 43 without GDM)	To assess the association between the serum concentration of adipokines and the development of GDM and to evaluate the circulation of these peptides at 24–28 weeks of pregnancy, prior to caesarean/vaginal delivery, 24 h after delivery, and within 2–6 months after delivery	Serum chemerin was significantly low in GDM (*p* = 0.02) and inversely associated with GDM (OR 0.85, 95%CI 0.73–0.98, *p* < 0.05). After adjustment for maternal age, gestation age, and BMI, the lowest tertile of the chemerin value was a strong predictor for GDM diagnosis (adjusted OR 4.5; 95% CI 1.4–14.0, *p* < 0.001).
Lee et al. (2021) [65]	South Korea	Humans	Prospective case–control study	65 women with previous GDM (20 with NGT vs. 23 with IGT and/or IFG vs. 22 with DM; groups were age- and BMI-matched)	To examine the relationship between the serial changes of adipokines (during pregnancy, 2 months after delivery, and 3 years after delivery) and the development of DM in women with GDM	Chemerin concentrations were not different among the three groups. No differences in chemerin concentrations were found 2 months after delivery or at 3 years postpartum among the three groups.
Mierzyński et al. (2021) [66]	Poland	Humans	Prospective case–control study	237 pregnant women (153 with GDM vs. 84 without GDM)	To evaluate serum chemerin, lipocalin 2, and apelin concentrations in GDM and healthy pregnant patients at 24–29 weeks of pregnancy	Chemerin concentration was significantly higher in the GDM group (*p* < 0.0001). The univariate linear regression model showed that for each 10 ng/mL increment of serum chemerin level, the GDM incidence increased by 18% (OR 1.180).
Bulut et al. (2021) [67]	Turkey	Humans	Prospective case–control study	51 pregnant women between 25 and 40 years and BMI < 30.0 (22 with GDM vs. 29 without GDM)	To evaluate the relationship between the 2nd and 3rd trimesters in salivary and blood levels of oxidative stress markers and chemerin in GDM and to study their correlations	Serum chemerin not associated with GDM (*p* = 0.412). No statistically significant association in salivary chemerin levels during 2nd and 3rd trimester between control and GDM (*p* = 0.466 and *p* = 0.530, respectively).
Fatima et al. (2021) [68]	Pakistan	Humans	Prospective case–control study	66 pregnant women between 18 and 40 years (33 with GDM vs. 33 without GDM)	To explore epigenetic modifications that may contribute to differential chemerin expression in maternal plasma (at 12th–14th weeks of gestation, 28th week of gestation, and 6th week postpartum), colostrum, and breast milk (at 6th week postpartum) and find their association with chemerin concentration in fetal cord blood and infant weight (at birth and 6 weeks postpartum)	Arterial cord blood chemerin higher in the GDM group (*p* = 0.004). Higher concentrations in the GDM group of colostrum chemerin (125.34 ± 15.88 ng/L vs. 24.97 ± 2.58 ng/L) and mature milk chemerin (177.40 ± 22.49 ng/L vs. 20.71 ± 2.36 ng/L). Colostrum and milk chemerin levels showed an independent association with infant weight at 6 weeks postpartum (r = 0.270, *p* = 0.034 and r = 0.464, *p* < 0.001, respectively) when adjusted for maternal BMI.
Wang et al. (2022) [69]	China	Humans	Prospective case–control study	703 women (303 pregnant women with GDM vs. 211 pregnant women without GDM vs. 189 nonpregnant healthy controls)	To examine the association of circulating chemerin levels (at 14–26th weeks of gestation) and genetic variants with GDM in a Chinese population	Plasma chemerin elevated in GDM patients when compared with nonpregnant controls (*p* < 0.0001) and no-GDM group (*p* < 0.0001). *RARRES2* variants (rs4721 and rs17173608) were associated with lower plasma levels of chemerin (*p* < 0.0001) and HOMA-IR (*p* < 0.0001) and protected against the development of GDM *p* < 0.0001).
Zhang et al. (2022) [70]	China	Humans and mice	Prospective case–control study	60 pregnant women (30 with GDM vs. 30 without GDM) and pregnant mice (streptozotocin-induced diabetic model vs. chemerin-induced diabetic model vs. controls)	To investigate the effect of placenta-derived exosomal miRNAs on fetoplacental endothelial dysfunction in GDM and to explore the role of chemerin	miR-140-3p and miR-574-3p were reduced in GDM patients (*p* < 0.01), resulting in abnormal proliferation, migration, and tube formation of umbilical vein endothelial cells. Chemerin was negatively associated with miR-140-3p and miR-574-3p levels (*r* = −0.712 and *r* = −0.728, respectively) in GDM.
Kaminski et al. (2023) [71]	Poland	Humans	Prospective case–control study	174 pregnant women (90 with GDM vs. 84 without GDM)	To evaluate serum adipokines levels measured at 24–29th gestational weeks in GDM	Chemerin significantly associated with GDM (*p* < 0.0001). Chemerin had a sensitivity of 66.67% and specificity of 89.29% in detecting GDM (AUC 0.767, 95%CI 0.697–0.827, *p* < 0.0001). One-way logistic regression analysis showed a 20% increase in the risk of GDM with a rise in chemerin concentration by 10 ng (OR 1.20, 95%CI 1.12–1.29, *p* = 0.0001).
Zhou et al. (2023) [72]	China	Humans	Prospective case–control study	50 pregnant women (25 with GDM vs. 25 without GDM)	To investigate the mechanism of the cGAS-STING signaling pathway (associated with mitochondrial dysfunction and IR) in GDM and its regulatory relationship with chemerin	In vitro, recombinant chemerin presented time-dependent inhibition on the cGAS-STING pathway on the insulin resistant cell model.
Chemerin and PE						
Xie et al. (2023) [73]	China	Humans	Meta-analysis	10 studies including 2130 pregnant women (832 with PE vs. 1298 without PE)	To evaluate the serum chemerin levels in women with PE	Positive correlation between elevated serum chemerin levels and PE diagnosis (MD 89.56 ng/mL, 95%CI 62.14–116.98 ng/mL; *p* < 0.001; I^2^ = 87%). Severe PE (MD 174.05 ng/mL, 95%CI 108.90–239.20; *p* < 0.001) was associated with a remarkable increment of serum chemerin as compared with mild PE (MD 67.89 ng/mL, 95%CI 25.64–110.14; *p* = 0.002; *p* value for subgroup difference 0.007).
Yin et al. (2023) [74]	China	Humans	Meta-analysis	13 studies including 2169 pregnant women (860 with PE vs. 1309 without PE)	To evaluate the serum chemerin levels in women with PE	Circulating chemerin levels were significantly higher in pregnant women with PE (SMD 1.39; 95%CI 1.02–1.77; I^2^ = 90.5%; *p* < 0.001). In subgroup analysis, chemerin levels were significantly increased in both Asian and non-Asian subgroups (Asia: SMD 1.59; 95%CI 1.08–2.10; I^2^ = 93.6%; *p* < 0.001; Others: SMD 0.98; 95%CI 0.72–1.25; I^2^ = 0.0%; *p* = 0.789). Significantly higher serum chemerin levels were correlated with disease gravity (mild PE: SMD 1.45; 95%CI 0.58–2.33; I^2^ = 94.4%; *p* < 0.001; severe PE: SMD 2.78; 95%CI 1.85–3.70; I^2^ = 92.3%, *p* < 0.001).
Chen et al. (2023) [75]	China	Humans	Prospective case–control study	620 pregnant women (310 with PE vs. 310 controls matched for maternal age, prepregnancy BMI and gestational age)	To examine the association between maternal serum chemerin levels at ≈35 gestational weeks and blood pressure in the postpartum period	Chemerin levels were significantly increased in PE (*p* < 0.01) and positively correlated with postpartum hypertension, defined as BP ≥130/80 mm Hg (r = 0.356, *p* < 0.001). Independent predictive role of a model including third-trimester maternal chemerin levels, systolic BP, HDL, LDL, triglycerides, fasting plasma glucose, primipara, delivery mode, and gestational age for postpartum hypertension after PE (AUC 0.903; 95% CI 0.869–0.937).
Tan et al. (2024) [76]	Netherlands	Humans	Prospective cohort study and prospective case–control study	467 pregnant women (cohort study) + 41 pregnant women (18 with early onset PE vs. 23 without PE) (case–control study)	To test serum chemerin as a marker for PE and the effect of statins on placental chemerin synthesis	Serum chemerin was associated with PE diagnosis even at early gestational stages (*p* < 0.01 at <29 weeks; *p* < 0.01 at 29–34 weeks; *p* < 0.001 at 35–42 weeks). Pravastatin and fluvastatin placental perfusion reduced chemerin placental upregulation in PE patients (both *p* < 0.01).
Ji et al. (2021) [77]	China	Rats	Prospective case–control study	24 pregnant rats (8 treated with chemerin vs. 8 treated with chemerin + α-NETA vs. 8 treated with saline solution)	To examine whether exogenous chemerin contributes to the pathogenesis of PE promoting macrophage polarization	Chemerin stimulated M1 macrophage polarization in a dose- and time-dependent manner, inhibited macrophage-induced trophoblast invasion and migration, and suppressed macrophage-mediated angiogenesis.
Tan et al. (2022) [78]	China	Humans and mice	Prospective case–control study	69 pregnant women (30 with PE vs. 29 without PE) and pregnant mice	To study placental chemerin expression and release in human pregnancy and the consequences of chemerin overexpression in both mice and immortalized human trophoblasts	Upregulation of placental chemerin synthesis in human and mice placental trophoblasts disturbed normal placental development via its CMKLR1 receptor, contributing to fetal growth restriction/resorption and the development of PE.
Tan et al. (2023) [79]	China	Mice	Prospective case–control study		To clarify if the high levels of chemerin released from placental trophoblasts might be a risk factor contributing to dyslipidemia during PE	Overexpression of placental chemerin production disrupted trophoblast lipid metabolism, potentially contributing to dyslipidemia in PE.
Bartho et al. (2023) [80]	Australia	Humans	Prospective case–control and prospective cohort study	1st cohort: 63 pregnant women (46 with early-onset PE vs. 17 without PE). 2nd cohort: 41 pregnant women (26 with PE vs. 15 without PE). 3rd cohort: 205 pregnant women (23 who developed PE/182 randomly selected controls).	To evaluate the biomarker potential of circulating chemerin to predict PE	Circulating chemerin was elevated in women with early-onset PE (*p* < 0.0006). Placenta chemerin expression was higher in women with early-onset PE (*p* < 0.0001). Serum chemerin was higher in established PE cohort than in controls (*p* = 0.006). Serum chemerin was higher in women who later developed PE than in controls (*p* < 0.0001).

AUC: area under the curve; BMI: body mass index; BP: blood pressure; cGAS-STING: cyclic GMP-AMP synthase stimulator of interferon genes; CI: confidence interval; CMKLR1: chemokine receptor-like 1; CRP: C reactive protein; DM: diabetes mellitus; GDM: gestational diabetes mellitus; GPR1: G protein-coupled receptor 1; HDL: high-density lipoprotein cholesterol; HOMA-IR: homeostasis model assessment–insulin resistance; IFG: impaired fasting glucose; IGT: impaired glucose tolerance; IR: insulin resistance; miRNA: micro ribonucleic acid; LDL: low-density lipoprotein cholesterol; MD: mean difference; mRNA: messenger ribonucleic acid; NETA: norethisterone acetate; NGT: normal glucose tolerance; OR: odds ratio; PE: preeclampsia; RARRES2: retinoic acid receptor responder 2; RT-PCR: real-time polymerase chain reaction; sFRP4: soluble frizzled-related protein 4; SMD: standard mean difference.

**Table 6:** Main interventional studies on chemerin in PCOS.

Reference	Country	Population	Study Design	Sample Size and Groups	Aim	Conclusions
Foda et al. (2019) [43]	Egypt	Humans	Prospective uncontrolled cross-sectional study	68 PCOS women (33 obese vs. 35 normal weight)	To study the effects of three months of metformin (starting by 500 mg once per day for one week, then 500 mg twice per day for the second week, followed by 500 mg three times a day to complete the three months) on serum chemerin in obese and normal-weight patients with PCOS	Metformin resulted in a significant decrease in serum chemerin levels in obese (*p* = 0.009) and normal-weight (*p* < 0.0001) patients with PCOS.
Pich et al. (2023) [81]	Poland	Rats	Nonblinded RCT	32 rats [16 with letrozole-induced PCOS (8 vitamin D-treated/8 untreated) vs. 16 controls (8 vitamin D-treated/8 untreated)]	To investigate the impact of 500 IU/day oral supplementation of vitamin D for 21 days on chemerin and adiponectin levels in the uteri of PCOS rats	Vitamin D supplementation decreased uterus chemerin levels to control levels (*p* < 0.05). Vitamin D also decreased CMKLR1 and GPR1 uterus levels (both *p* < 0.05), but increased CCRL2 uterus level (*p* < 0.05), in PCOS rats. In control animals, vitamin D did not change plasma levels of chemerin.
Saraf-Bank et al. (2019) [82]	Iran	Humans	Double-blinded RCT (computer-generated randomization according to weight, height, BMI, age and gender)	60 adolescent women with BMI > 25 kg/mq (30 patients who received intervention vs. 30 patients who received placebo)	To evaluate the effects of 500 mg/day curcumin supplements for 10 weeks on inflammation, oxidative stress, and serum chemerin levels in overweight and obese patients	No significant changes in chemerin levels after curcumin supplementation (*p* = 0.749; *p* = 0.808 adjusted for age and BMI, respectively).
Pouteymour Fard Tabrizi et al. (2020) [83]	Iran	Humans	Double-blinded RCT (computer-generated randomization according to age and BMI)	48 women with PCOS between 20 and 45 years old and BMI 30–40 kg/mq (24 intervention group vs. 24 placebo)	To evaluate the effects of 5 g/day thylakoid-rich spinach extract supplementation combined with a hypocaloric diet for 12 weeks on BMI and serum adipokine levels	Thylakoid-rich spinach extract combined with a low-calorie diet decreased circulating chemerin (*p* < 0.001, adjusted for baseline values, duration of PCOS, and the changes in calorie intake and physical activity level at the end of the study).

BMI: body mass index; CCRL2: C-C motif chemokine receptor-like 2; CMKLR1: chemokine receptor-like 1; GPR1: G protein-coupled receptor 1; PCOS: polycystic ovary syndrome; RCT: randomized controlled trial.

**Table 7:** Meta-analyses on the association between chemerin levels in women with and without PCOS.

Reference	Studies (n.)	Patients Studied (n.)	Results	Aim	Conclusions
Lin et al. (2021) [20]	5 (serum)	414 (219 PCOS vs. 195 no-PCOS)	SMD ^1^ 1.13; 95%CI 0.08 to 2.18; I^2^ = 96%; *p* = 0.03	To assess whether the levels of circulating adipokines are changed in nonobese PCOS	PCOS was significantly associated with an increased chemerin level. However, the CI revealed no significant difference between PCOS and controls (unit included in the CI range).
Mansoori et al. (2021) [36]	19 (serum)	2256 (1191 PCOS vs. 1065 no-PCOS)	WMD ^1^ 12.02 pg/mL; 95%CI 10.92 to 13.13; I^2^ = 99.4, *p* < 0.001	To compare serum and FF chemerin and ovarian chemerin mRNA expression between women with and without PCOS	Serum and FF chemerin and mRNA expression were higher in the PCOS patients.
	4 (FF)	224 (114 PCOS vs. 110 no-PCOS)	WMD ^1^ 41.7 pg/mL; 95%CI 17.89 to 65.5; I^2^ = 83.5%, *p* < 0.001		
	3 (mRNA expression)	204 (104 PCOS vs. 100 no-PCOS	WMD ^1^ 0.38%; 95%CI 0.25 to 0.52; I^2^ = 82.8, *p* = 0.001		
Mehrabani et al. (2021) [37]	14 (serum)	1605 (913 PCOS vs. 692 no-PCOS)	SMD ^1^ 1.87; 95%CI 1.35 to 2.40; I^2^ = 94.4%, *p* < 0.001	To study the circulating concentration of adipokines in PCOS subjects	Serum levels of chemerin were significantly higher in PCOS subjects than in controls.
Gao et al. (2023) [38]	9 (serum)	1240 (746 PCOS vs. 494 no-PCOS)	SMD ^1^ 0.79; 95%CI 0.36 to 1.23; I^2^ = 91.7%, *p* < 0.01	To evaluate the apelin and chemerin levels of patients with PCOS	The circulating chemerin levels in patients with PCOS were significantly higher than those in controls.

BMI: body mass index; CI: confidence interval; FF: follicular fluid; mRNA: messenger ribonucleic acid; PCOS: polycystic ovary syndrome; SMD: standard mean difference; WMD: weighted mean difference. ^1^. SMD and WMD were used if the meta-analyses assessed results with different units of measurement or with the same unit of measurement, respectively.
